# Supplementary material for: ProtFun: a protein function prediction model using graph attention networks with a protein large language model
Source: Bioinform Adv. 2025 Oct 11;5(1):vbaf245. doi: 10.1093/bioadv/vbaf245 (PMC12571506; doi:10.1093/bioadv/vbaf245)
Supplement: vbaf245_Supplementary_Data [file vbaf245_supplementary_data.pdf]

ProtFun: A Protein Function Prediction Model Using Graph Attention Networks with a Protein Large Language Model

Muhammed Talo and Serdar Bozdag

Supplementary Document

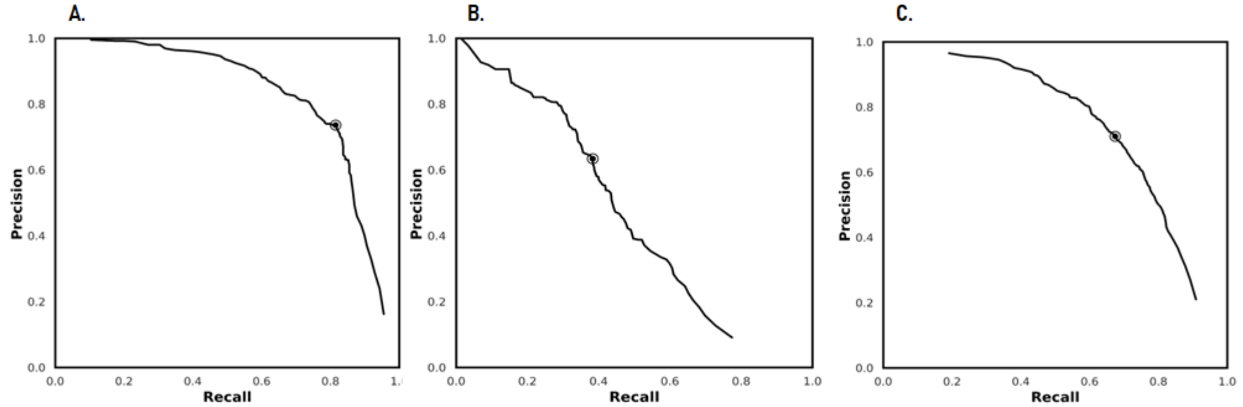

Fig S1. Precision-recall performance values of the ProtFun model for human in MFO (A), BPO (B), and CCO (C) subontologies on the NetGO dataset.

Table S1. Performance of the model on human, mouse, and *Arabidopsis thaliana* utilizing the DeepGOZero dataset.

	F_{max} (higher is better)			S_{min} (lower is better)			**AUPRC** (higher is better)		
Species (Taxon ID)	**MFO**	**BPO**	**CCO**	**MFO**	**BPO**	**CCO**	**MFO**	**BPO**	**CCO**
Human (9606)	0.679	0.505	0.721	7.406	43.472	8.069	0.676	0.493	0.631
Mouse (10090)	0.673	0.449	0.691	6.491	52.155	7.203	0.667	0.425	0.531
Arabidopsis thaliana (3702)	0.782	0.461	0.750	3.377	24.795	5.434	0.777	0.420	0.631

Table S2. Performance comparison of the ProtFun model on CAFA species utilizing the NetGO dataset.

	F_{max} (higher is better)			S_{min} (lower is better)			**AUPRC** (higher is better)		
Taxon ID	**MFO**	**BPO**	**CCO**	**MFO**	**BPO**	**CCO**	**MFO**	**BPO**	**CCO**
9606	0.799	0.531	0.696	2.722	23.185	6.676	0.805	0.495	0.617
3702	0.632	0.459	0.739	4.830	27.293	4.475	0.609	0.372	0.626
6239	0.735	0.442	0.723	4.814	33.092	3.184	0.769	0.396	0.607
7227	0.611	0.532	0.747	4.627	20.605	5.258	0.576	0.498	0.634
7955	0.671	0.363	0.706	5.831	25.936	6.107	0.626	0.290	0.433
9823	0.739	0.504	0.778	3.877	20.518	4.000	0.643	0.473	0.599
10090	0.627	0.410	0.659	5.381	44.610	10.717	0.615	0.363	0.556
10116	0.761	0.495	0.737	3.172	32.903	4.798	0.753	0.430	0.632
44689	0.747	0.392	0.705	3.462	16.158	5.516	0.652	0.316	0.637
284812	0.616	0.514	0.697	3.936	13.032	6.451	0.507	0.507	0.567
559292	0.640	0.441	0.751	3.350	21.991	6.325	0.460	0.425	0.639
